# Apolipoprotein E (APOE) Haplotypes in Healthy Subjects from Worldwide Macroareas: A Population Genetics Perspective for Cardiovascular Disease, Neurodegeneration, and Dementia

**DOI:** 10.3390/cimb45040184

**Published:** 2023-03-31

**Authors:** Paolo Abondio, Francesco Bruno, Donata Luiselli

**Affiliations:** 1 Laboratory of Ancient DNA, Department of Cultural Heritage, University of Bologna, Via degli Ariani 1, 48121 Ravenna, Italy; 2 Laboratory of Molecular Anthropology and Center for Genome Biology, Department of Biological, Geological and Environmental Sciences, University of Bologna, Via Selmi 3, 40126 Bologna, Italy; 3 Regional Neurogenetic Center (CRN), Department of Primary Care, ASP Catanzaro, 88046 Lamezia Terme, Italy; 4 Association for Neurogenetic Research (ARN), 88046 Lamezia Terme, Italy

**Keywords:** apolipoprotein E, cardiovascular disease, dementia, neurodegeneration, Alzheimer’s disease, population genetics, mutation, haplotype, public health, medicine

## Abstract

Human APOE is a 299-amino acid long protein expressed and secreted in several tissues and body districts, where it exerts different functions mainly related to lipid metabolism, with specific activities around cholesterol transport and absorption/elimination. It has three main isoforms, determined by the pair of mutations rs7412-C/T and rs429358-C/T, which gives rise to the functionally different APOE variants ε2, ε3, and ε4. These have a distinct impact on lipid metabolism and are differentially implicated in Alzheimer’s disease and neurodegeneration, cardiovascular disease, and dyslipidemia. A plethora of other single nucleotide variants along the sequence of the APOE gene have been studied in cohorts of affected individuals, where they also modulate the influence of the three main isoforms to determine the risk of developing the disease. However, no contextual analysis of gene-long haplotypes has been carried out so far, and never extensively in cohorts of healthy individuals from different worldwide populations. Leveraging a rich population genomics dataset, this study elucidates the distribution of APOE variants and haplotypes that are shared across populations and to specific macroareas, revealing a variety of risk-allele associations that distinguish specific ancestral backgrounds and can be leveraged for specific ancestry-informed screenings in medicine and public health.

## 1. Introduction

Human APOE is a 299-amino acid long protein in its mature form (317 residues in the unprocessed form) that is expressed and secreted chiefly by monocytes/macrophages, astrocytes, adipose tissue, and liver cells [1,2,3]. Its functional domains include an N-terminal low-density-lipoprotein receptor (LDLR) binding region and a hydrophobic C-terminal domain that mediates lipid binding and oligomerization [4,5]. The combination of two mutations at the APOE gene (rs7412-C/T and rs429358-C/T) originates the three main protein variants, called ε2, ε3, and ε4 (or, alternatively, APOE2, APOE3 and APOE4). Isoform ε3 has a cysteine in position 130 and an arginine residue in position 176 (of the immature form), while isoform ε4 has two arginine residues and isoform ε2 has two cysteine residues [5,6]. Several other mutations can act on this background to modulate the effects of these isoforms and are involved in several neurodegenerative and cardiovascular pathologies [7,8]. APOE isoforms mediate multiple functions associated with lipid metabolism: ε3, for example, maintains the structural integrity of lipoproteins, enhances the solubilization of cholesterol in blood plasma, regulates lipid homeostasis, and activates the reverse cholesterol transport, redirecting fatty acids to the liver for degradation [4,9]. The APOE genotype also accounts for most of the risk related to Alzheimer’s disease development, with ε4 being the most involved isoform in this case [3]. APOE isoforms can also heavily interfere with other risk factors for cardiovascular diseases (CVD). For example, fasting insulin represents a cardiovascular risk only in subjects carrying a copy of the ε2 allele, while ε4 allele is a risk factor for the onset of type I diabetes mellitus, as well as a predisposing factor for the development of lipoprotein glomerulopathy and diabetic nephropathy [10,11,12]. Extensive genetic studies on these isoforms also associate APOE isoforms with well-being and longevity [13]: individuals with the ε3 allele gain more energy from food and body fat deposits, while subjects carrying ε4 undergo rapid fatty acid mobilization associated with a high-fat diet in colder climates [14,15]. Indeed, it is well known that APOE is a pleiotropic gene, its protein isoforms have different functions in several expression contexts, as well as that the environment is crucial for the manifestation of a pathological condition [16,17]. However, there are few studies trying to observe this gene beyond the three classic isoforms and the simple effect of single point mutations across its length in the context of cardiovascular and neurological diseased states [18,19]. Moreover, extensive epidemiological and genomic studies have most often been performed on subjects of European or mixed African ancestry in the USA, with a remarkable lack of specific genetic information from underrepresented populations in the Asian and South American geographic regions. This cohort bias hinders the overall understanding of the impact of genetic mutations, haplotypic variation and their phenotypic manifestations in the context of cardiovascular disease, neurodegeneration, and dementia.

Given these premises, the present study aims at detecting the presence of 68 single-point mutations along the sequence of the *APOE* gene in a publicly available dataset (identified as the “1KGP dataset”) including the genetic sequences of 2504 healthy individuals from 26 worldwide populations in five macroareas. All mutations detected in the dataset are analyzed for their distribution across populations and macroareas. Moreover, haplotype reconstruction and the definition of population- and macroarea-related haplotype sequences are used to explore the variability of disease-associated mutations that could point to specific cross-population and cross-ancestry admixture events.

## 2. Materials and Methods

### 2.1. Data Recovery and Management

The present study makes use of the freely accessible datasets produced by the 1000 Genomes Project Consortium (1KGP) [20]. In particular, the variant call format (VCF) file for chromosome 19, where the APOE gene is located, has been downloaded [21]. The VCF file contains genetic information for over a million single nucleotide variants (SNVs), from which only those falling into the length of the gene of interest were recovered after quality control (see Section 2.2) and haplotype estimation (see Section 2.3) for subsequent analysis. Moreover, 2504 unrelated, self-reportedly healthy individuals (out of 3202 subjects) over the age of 18 years were extracted from the same dataset, as representatives of 26 populations of diverse local ancestry and genetic variability in five geographic macroareas (Africa, AFR, 661 individuals; America, AME, 347 individuals; Europe, EUR, 504 individuals; East Asia, EAS, 503 individuals; and South Asia, SAS, 489 individuals). The remaining 698 individuals were excluded from the analysis, because they represent first or second-degree relatives and their genetic similarity could skew the results towards kinship-driven heredity, instead of the genetic randomness one would expect when approximating a large, open, panmictic population.

A total of 68 unique polymorphic single nucleotide variants (SNVs) in the sequence of APOE, included between the gene starting and ending points at physical positions 44,905,791 and 44,909,393, respectively, along the sequence of chromosome 19, were recovered (this information was extracted from the Ensembl genome browser [22] release 109, dated February 2023, at http://www.ensembl.org/Homo_sapiens/Gene/Summary?db=core;g=ENSG00000130203;r=19:44905791-44909393, and last accessed on 9 February 2023). Their association with known physiological and pathological phenotypes, as well as their clinical significance, have been checked through repository research in the GWAS Catalog (https://www.ebi.ac.uk/gwas/, last accessed on 9 February 2023), AlzForum (https://www.alzforum.org/mutations/apoe, last accessed on 9 February 2023), and NIH ClinVar (https://www.ncbi.nlm.nih.gov/clinvar/, last accessed on 9 February 2023), and confirmed through corresponding peer-reviewed scientific literature. Pairwise linkage disequilibrium (LD) among variants has been recovered by parsing the 68 mutations in the LDMatrix web-based application (https://ldlink.nci.nih.gov/?tab=ldmatrix, last accessed on 9 February 2023), which estimates LD on the basis of the 1KGP dataset itself, so it provides a realistic match to the dataset in use. Only variants with an r^2^ (correlation coefficient) value of LD greater than 0.8 are considered significantly in linkage.

### 2.2. Dataset Quality Control and Linkage Estimation

A limited quality control (QC) procedure was performed on the complete 1KGP dataset, which includes almost 80 million single nucleotide variants covering all autosomal chromosomes (therefore, excluding the X and Y chromosomes, as well as mitochondrial DNA). This was performed before extracting variants limited to chromosome 19, where the gene of interest is located, so as not to introduce any bias. QC was performed with the PLINK version 1.9 software (https://www.cog-genomics.org/plink/, last accessed on 9 February 2023) [23] after conversion of the original vcf files in PLINK format through the PLINK software itself (using the input command --vcf and requesting a converted output with --make-bed) and included the removal of variants or individuals with data missingness greater than 1% (--geno 0.01; --mind 0.01). A check for the respect of Hardy–Weinberg equilibrium for each variant was also performed, applying a Bonferroni correction for multiple testing to the standard threshold of 0.01, which is then divided by the number of variants (--hwe 1.8 × 10^−10^). All remaining variants on chromosome 19 were extracted (--chr 19) for haplotype reconstruction.

### 2.3. Data Phasing and Haplotype Estimation

In order to assess the presence of APOE mutations in the context of the surrounding variants, to detect finer-scale relationship patterns and to compare chromosomal sequences for further statistical analysis, a data phasing procedure and haplotype estimation were performed in order to define which allele of every variant is located on which copy of the chromosome, for each of the 2504 individuals of the 1KGP dataset. To do so, ambiguous SNVs (carrying an A/T or C/G combination of alleles, for which the chromosome copy and strand cannot be univocally defined) were concealed by temporarily changing them into pairs of non-complementary alleles for the sake of conserving crucial variants along the length of the APOE gene sequence (they have been reverted to their original identity after phasing). Moreover, information about the ancestral or derived nature of each variant was deduced by using a reconstructed reference human genome sequence as a guide for distinguishing between ancestral and derived alleles. In particular, the ancestral/derived state of each allele in such a reference sequence was previously assigned by aligning it with the Ensembl Compara 6 primates EPO genome sequences [24], and only alleles present in all the compared genomes were considered as ancestral. Haplotype estimation is finally performed using the SHAPEIT software version 1.9 (https://mathgen.stats.ox.ac.uk/genetics_software/shapeit/shapeit, last accessed on 9 February 2023) [25,26] with default parameters settings and the HapMap phase 3 recombination map for chromosome 19.

## 3. Results

### 3.1. Variant Summary Statistics

As reported in Supplementary Table S1, of the 68 polymorphic point mutations that could be recovered along the sequence of the APOE gene for the phased dataset of the 1000 Genomes Project (human reference build hg38), a total of 10 variants (rs373985746, rs440446, rs192348494, rs150375400, rs769449, rs769450, rs201672011, rs370594287, rs121918393, and rs769455) were explicitly linked to modulating the risk of Alzheimer’s disease and other neurodegenerative disorders (14.7% of the total SNPs), while nine (rs769448, rs61357706, rs115299243, rs1018669382, rs72654468, rs530010303, rs140808909, rs374329439, and rs553874843) were associated with the risk of cardiovascular ailments (13.2% of the 68 polymorphisms) and six other variants (rs533904656, rs769452, rs429358, rs7412, rs199768005, and rs190853081) have been involved in both neurodegeneration and cardiovascular distress (8.8% of the considered point mutations). The other 32 variants (47%) have not been linked to pathological states so far. Now, if a threshold of 0.01 for minor allele frequency (MAF) is taken into consideration to define variant rarity over the whole dataset, then 61 out of 68 (or 89.7%) of the objects considered are rare: the only common variants are rs440446 (MAF = 0.374), rs877973 (0.016), rs565782572 (0.012), rs769449 (0.065), rs769450 (0.328), rs429358 (0.149), and rs7412 (0.075). Of these, rs877973 and rs565782572 have not been linked to neurodegeneration or cardiovascular disease, while rs440446, rs769449, and rs769450 are chiefly involved in AD, and the combination of alleles carried by variants rs429358 and rs7412 concur to define the three main isoforms of the APOE protein: ε2 (mostly protective against neurodegeneration, but involved in cerebral amyloid angiopathy, CAA), ε3 (the “wild-type”, or most common isoform worldwide), and ε4 (most probably the ancestral isoform, chiefly linked to Alzheimer’s and cardiovascular disease).

### 3.2. Variant Uniqueness and Genotype Distribution across Macroareas

Variant uniqueness can be defined as the presence of a specific variant in a polymorphic state only in one of the five considered macroareas. According to this definition, the South Asian macroarea (SAS) has the most unique variants, with 12 or 17.6% of the total (rs555840707, rs555877419, rs550501196, rs555914310, rs552962455, rs572713679, rs542186645, rs529662056, rs563103121, rs555222732, rs774452222, and rs569017773); they are all rare, according to the definition provided in the previous Section 3.1, and none has been linked to cardiovascular ailments or neurodegeneration before (Table 1 and Supplementary Table S2). Interestingly, two variants only found in two individuals in this macroarea (rs552962455 and rs572713679) are located in consecutive positions along the second intron of the APP gene and are in complete linkage (r^2^ = 1).

The African macroarea (AFR) comes next, with 11 unique variants present only in African populations, or 16.2% of the total (rs72654467, rs1187843706, rs184686013, rs375741166, rs189660912, rs564144591, rs148558158, rs1368528953, rs577618688, rs1018669382, and rs1181840153). These are all rare, including one (rs1018669382) already identified in one French patient diagnosed with dyslipidemia from a cohort of 6000 subjects [Abou Khalil]. Moreover, if we take into consideration variants present in a polymorphic state in AFR for at least 80% of the observations, seven more SNPs also emerge (and they are also found only in people of Central American ancestry). Four of these (rs769453, rs877973, rs545943117, and rs9282609) have not been linked to disease, while the remaining three are found in high linkage disequilibrium (r^2^ > 0.8) and in a total of 33 individuals of African ancestry: rs61357706, rs115299243, and rs769455, with the last one already studied in the context of neurodegeneration with mixed results (because of its overall rarity, no statistical support could be gained for a link with AD dementia), but known for being a risk factor for hyperlipoproteinemia type III (HLPP3) in American individuals of African ancestry [Koopal, Medway]. The other two variants have also already been linked to cardiovascular risk (Table 1 and Supplementary Table S2) [27,28,29,30].

The East Asian macroarea (EAS) is characterized by nine unique variants (rs373985746, rs192348494, rs150375400, rs373651604, rs549553647, rs533904656, rs140808909, rs190853081, and rs553874843), of which only two (rs373651604 and rs549553647) have not been associated with neurodegeneration or cardiovascular disease. Furthermore, three East Asian individuals are the only carriers of polymorphisms in variants rs140808909 and rs190853081: both mutations are located in exon 4, where they induce an aminoacidic change from glutamic acid (Glu, or E) to lysin (Lys, or K) in two consecutive positions along the resulting protein (residues 182 and 183, respectively); both are related to cardiovascular impairment, and both are characterized by high values of linkage disequilibrium (r^2^ > 0.8), so they tend to be inherited together more often than by chance (Table 1 and Supplementary Table S2).

The American macroarea (AME) is characterized by seven unique variants (rs528229851, rs538246559, rs539807928, rs535397097, rs1227709957, rs1313313298, and rs557715042), none of which has been linked to disease risk in the case of neurodegeneration or cardiovascular ailments. Mutations rs1227709957 and rs1313313298, found in a single individual, are in high linkage disequilibrium (r^2^ > 0.8) and are located in exon 4 of the APOE gene (Table 1 and Supplementary Table S2).

Finally, six unique variants for the European macroarea (EUR) are detected (rs563571689, rs769447, rs769452, rs186466504, rs121918393, and rs530010303). Three of these (rs563571689, rs769447, and rs186466504) have not been linked to pathological conditions, while the other three have been studied in the context of neurodegeneration and cardiovascular disease (Table 1 and Supplementary Table S2).

These observations also imply that only six polymorphic point mutations are found across all five macroareas: rs440446, rs565782572, rs769449, rs769450, rs429358, and rs7412. These variants have already been described in Section 3.1, as these are also six of the seven variants that are not considered “rare” by definition in our dataset (MAF > 0.01). The remaining variant, rs877973, is present in 76 individuals of African ancestry and 3 people of American descent. It is also interesting to notice that variant rs769449, although present in all macroareas, is contributed to the AFR group from populations that may be considered “admixed” and “*in diaspora*” (meaning, in this case, that assigned ancestry and geographical location of the sampled individuals do not necessarily match), as this mutation only appears in nine subjects collected from either individuals of African ancestry in South-Western USA, or individuals of African ancestry in the Caribbean, but not in the other five populations that have been collected locally across the African continent (Table 1 and Supplementary Table S2).

### 3.3. APOE Isoform Frequency across and within Macroareas

**Figure 1:**
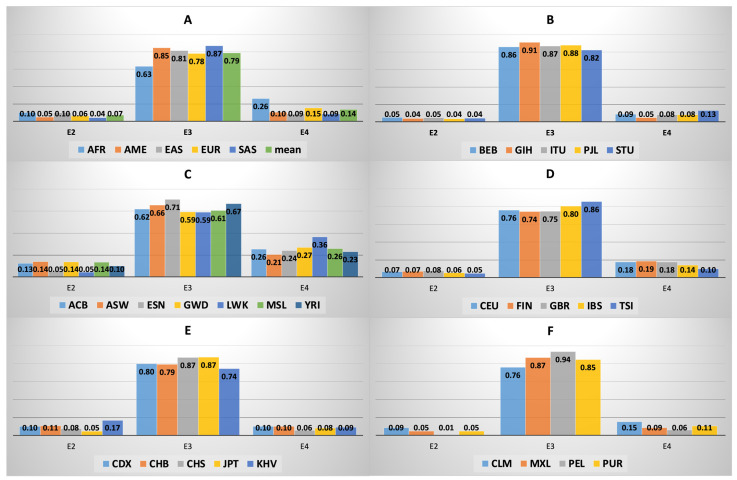
Distribution of APOE ε2, ε3 and ε4 isoforms: (**A**) across macroareas; (**B**) across the South Asian populations; (**C**) across the African populations; (**D**) across the European populations; (**E**) across the East Asian populations; (**F**) across the American populations.

A frequency analysis of APOE ε2, ε3, and ε4 isoforms has been performed across macroareas (Figure 1 and Supplementary Table S3). The ε3 isoform is present in the dataset at an average frequency of 0.79 (with a minimum frequency of 0.63 in the AFR macroarea and a maximum frequency of 0.87 in the SAS macroarea); ε4 has a dataset frequency of 0.14, with a minimum of 0.09 (in the SAS and EAS macroareas) and a maximum of 0.26 in the AFR macroarea; ε2 has a more uniform distribution, with an average frequency of 0.07, a minimum value of 0.04 in the SAS macroarea, and a maximum of 0.10 in the AFR macroarea. The general distribution of APOE isoforms across macroareas provides a significant value when analyzed through a Chi-square test (df = 8, *p*-value = 1.92 × 10^−54^), suggesting that the observed distribution is significantly different than its expected counterpart. If the average worldwide frequency is taken into consideration as the expected frequency values for each single macroarea separately, then the Chi-square test reveals that the EUR population does not, indeed, differ in observed and expected distributions, while the other macroareas are significantly different (AME: *p*-value= 5.83 × 10^−4^; EAS: *p*-value= 1.74 × 10^−7^; SAS: *p*-value= 8.71 × 10^−9^; AFR: *p*-value = 6.31 × 10^−46^; df = 2 for all tests), with the African macroarea contributing to most of the overall significance. When focusing on the distribution of APOE isoforms within macroareas and a Chi-square test is performed, only the populations in the AFR macroarea provide a marginally significant result, with the Luhya (LWK) people highlighted as the only contribution towards significance (df = 2, *p*-value = 0.026), since this group presents a lower number of individuals with the ε2 and ε3 isoforms than what is expected, and a higher number of individuals with the ε4 isoform of APOE (Figure 1 and Supplementary Table S3).

### 3.4. APOE Core Haplotype Characteristics

**Figure 2:**
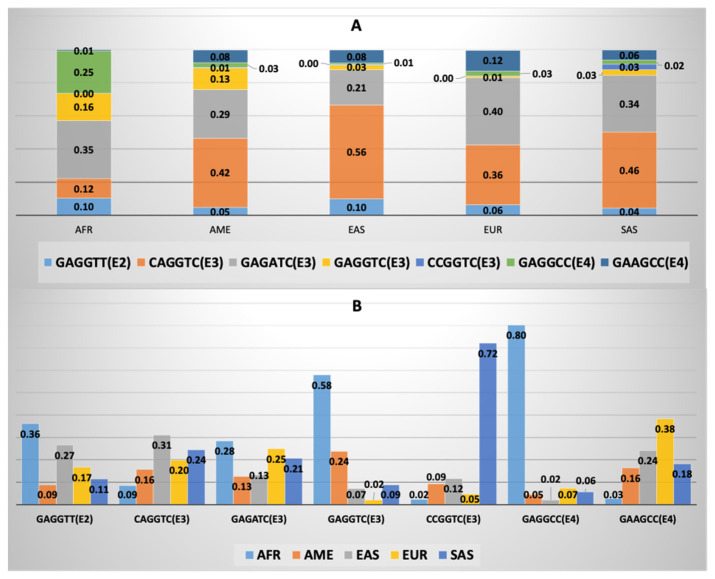
Core haplotype frequencies across macroareas. (**A**) Distribution of haplotype frequencies in each macroarea: (**B**) proportion of contribution from each macroarea to a specific haplotype.

The six polymorphic point mutations that are found across all five macroareas (rs440446, rs565782572, rs769449, rs769450, rs429358, and rs7412) compose a shared cluster of variants that can be traced through their allelic composition. We can define this as a core haplotype, because at least one individual in each macroarea carries the mutated allele for one of the variants. Then, this allows for a comparison of each six-allele sequence across macroareas (Figure 2 and Supplementary Table S4).

It is interesting to notice that, even though six polymorphic biallelic variants could theoretically produce 2^6^ = 64 different combinations, only 14 of them arise worldwide, with just seven of them having a frequency of at least 0.01 in at least one population, making them non-rare. Conversely, the remaining seven existing combinations are rare across all macroareas, with four of them being present only once overall. When looking at APOE isoforms, of these fourteen haplotypes, only one carries isoform ε2 (G-A-G-G-T-T, where the isoform is represented by the last two bases), while eight carry ε3, and five contain isoform ε4. However, if we focus on the seven non-rare haplotypes, then only four of them are associated with isoform ε3 and only two to ε4 (Figure 2 and Supplementary Table S4). None of these sequences seem to carry more than two disease-linked variants (excluding APOE isoforms).

It is evident that the African individuals are major contributors to the G-A-G-G-C-C (ε4) combination (which makes up 25.5% of the total AFR macroarea haplotypes), with 80.2% of its worldwide presence ascribable to them. Conversely, they marginally participate (2.8%) to the presence of the other ε4 haplotype, G-A-A-G-C-C, which is principally contributed by the EUR (38.4%) and EAS (24.1%) macroareas. The latter group also shows a major presence (31.2%) in the ε3 haplotype C-A-G-G-T-C (which does not carry causative mutations), as well as the ε2 haplotype G-A-G-G-T-T (26.7%), right behind the AFR macroarea (36.3%).

**Figure 3:**
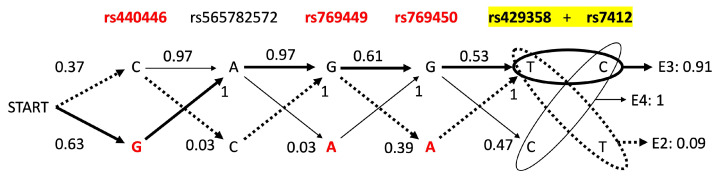
Step-by-step frequency graph for the core haplotypes. AD-related variants (and the specific risk allele) are highlighted in red; variants contributing to APOE isoforms are highlighted in yellow. The most frequent theoretical haplotype is indicated by solid, bold arrows and circles; the least frequent theoretical haplotype is indicated by dashed arrows and circles.

If a simple graph is built, where each successive node is one of the two possible alleles for every variant, and each edge carries the probability of going from one node to the next (where the probability is given by the frequency of the observed change in allele from one variant to the next), it can be easily seen that the edges with the highest probability (*p* = 1) are the ones stemming from an AD-related causative allele and connecting to the non-damaging allele of the next variant (Figure 3). Vice versa, the edges with the lowest probability are those going towards a causative allele (as low as 0.01), apart for the starting variant, which has a much higher probability for the damaging allele G, rather than its counterpart C. Indeed, the path with the highest probability produces the haplotype G-A-G-G-T-C, leading to an APOE ε3 isoform, although in our dataset, this combination of variants is not the most abundant by far (Figure 3).

### 3.5. APOE Super-Core Haplotype Characteristics

**Figure 4:**
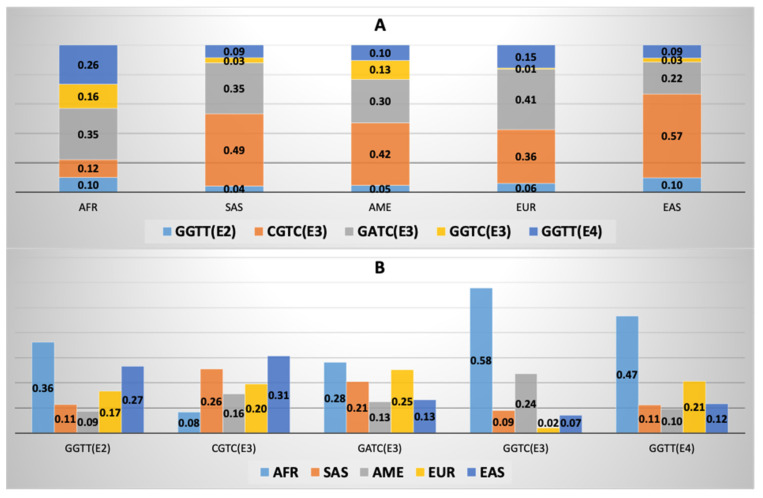
Super-core haplotype frequencies across macroareas. (**A**) Distribution of haplotype frequencies in each macroarea: (**B**) proportion of contribution from each macroarea to a specific haplotype.

If the attention is focused specifically on variants that are polymorphic at least once in every single population of the 1KGP dataset, then only four variants emerge (rs440446, rs769450, rs429358, and rs7412) that compose a sub-cluster of the SNPs identified as shared across macroareas in the previous Section 3.4. These four variants carry alleles involved in the neurodegenerative and cognitive alteration of the individual (especially rs440446-G and rs769450-A), with an overlapping layer of cardiovascular and cerebrovascular ailments deriving especially from the ε2 and ε4 isoforms. Indeed, out of 2^4^ = 16 possible combinations, there is only one haplotype carrying APOE ε2, while four carry isoform ε3 and three contain ε4 (Figure 4 and Supplementary Table S5). Three of the combinations, however, appear only once in the dataset: C-A-T-C (ε3 carrying the risk allele rs769450-A) in a Chinese Han individual from Beijing (CHB); G-A-C-C (ε4 and carrying both the causative mutations mentioned above) in a Mende, Sierra Leone sample (MSL); and C-G-C-C (ε4 but carrying none of the other neurodegeneration-associated mutations) in a South Asian individual from Bangladesh (BEB). Considering the remaining five combinations and their distribution across macroareas, it can be noticed how the AFR group exhibits a higher proportion of the ε2- and ε4-carrying haplotypes (10.3% and 26.3% of the overall AFR haplotypes, but they contribute to 26.3% and 46.6% of the respective haplotypes), and it provides more than half of the total ε3-carrying haplotype G-G-T-C found in the dataset, with 217 copies against a total of 375, or 57.9% (the AME macroarea is the second highest contributor, with 89 copies or 23.7%). On the contrary, the EUR macroarea seems to be particularly devoid of this specific sequence, as it appears only eight times overall; however, it is a significant contributor to another ε3 haplotype which carries both mutations associated with neurodegeneration, G-A-T-C (25.2%, just behind AFR with 28.2%), as well as the ε4-carrying sequence, G-G-T-T, with 20.8% of them coming from individuals of European ancestry. East Asian and South Asian populations particularly contribute to the ε3-including haplotype C-G-T-C (30.7% and 25.6% respectively), which does not carry any risk allele; indeed, this sequence makes up 48.9% of the South Asian and 56.9% of the East Asian total haplotypes (Figure 4 and Supplementary Table S5).

**Figure 5:**
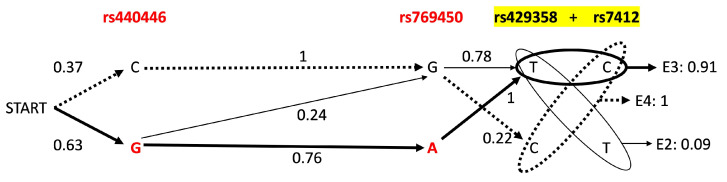
Step-by-step frequency graph for the super-core haplotypes. AD-related variants (and the specific risk allele) are highlighted in red; variants contributing to APOE isoforms are highlighted in yellow. The most frequent theoretical haplotype is indicated by solid, bold arrows and circles; the least frequent theoretical haplotype is indicated by dashed arrows and circles. Variants have been located in the same positions as Figure 3 to facilitate comparison.

If a simple step-by-step occurrence graph of the five common haplotypes is built as described in Section 3.4, one can notice that, having eliminated two alleles from the previously described six-variant haplotype, the situation has reversed: if the haplotype starts with the neutral allele rs440446-C, then the second position will always be occupied by another neutral allele, rs769450-G, while if the sequence starts with the causative allele, rs440446-G, then the second position will be taken by the also damaging allele rs769450-G 76% of the time, and the carried isoform will most probably be APOE ε3 (Figure 5).

### 3.6. APOE Macroarea-Specific Haplotypes

If we take into consideration variants that are polymorphic at least once in each population for a given macroarea, but are not necessarily so in all other macroareas, then five separate macroarea-specific haplotypes can be determined (Supplementary Table S6).

Indeed, for the South Asian cohort (SAS), the variants composing their specific haplotype are the same that were used to define the six-variant macroarea-level haplotype in Section 3.4 (rs440446, rs565782572, rs769449, rs769450, rs429358, and rs7412), meaning that all these SNPs appear as polymorphic at least once in each single SAS population, but not in all populations from the other macroareas. Indeed, since we have also defined the more stringent population-level haplotype in Section 3.5, which is composed of four variants (rs440446, rs769450, rs429358, and rs7412) that are present at least once in all populations and will, therefore, appear in all core population haplotypes as well, we can immediately deduce that the two extra variants in the case of the SAS macroarea are rs565782572 and rs769449. A total of 11 different haplotypes emerge, with one carrying the APOE ε2 isoform variants (G-A-G-G-T-T), six carrying ε3 (but three of these are rare in the South Asian group), and four carrying ε4 (two being rare). The two most represented haplotypes (both ε3) are C-A-G-G-T-C, which does not include any disease risk-modifying allele, and G-A-G-A-T-C, which harbors two mutations related to a higher risk of developing neurodegeneration (rs440446-G in first position and rs769450-A in fourth position).

The European populations are characterized by five variants, instead of six: rs440446, rs769449, rs769450, rs429358 and rs7412 (Supplementary Table S6). Their polymorphic status gives rise to six haplotypes, of which one presents the terminal alleles for the ε2 isoform, three the ε3 combination (but one of them is a rare haplotype with frequency below 0.01), and two present ε4. The only disease-related variant in this case is rs769449-A, which is found only in the ε4-bearing haplotype G-A-G-C-T and has a frequency of 12.3% in the EUR macroarea.

The American macroarea carries the same five variants identified for the EUR group, with the inclusion of an extra polymorphism, rs769451, that has not been associated with any increased risk of neurological or cardiovascular disease so far. Indeed, the minor allele rs769451-G itself is quite rare, appearing only five times and being linked to two ε3-bearing haplotypes, which of course become rare in the whole population (Supplementary Table S6). Other than these, the AME macroarea presents one ε2-carrying haplotype, two more common sequences with ε3 terminal alleles, and two ε4 haplotypes. If we exclude the rare polymorphism, then we obtain for the AME group the same haplotypes of the EUR macroarea, with slightly different overall frequencies. The most striking one concerns an enrichment in the American populations of the ε3-bearing haplotypes G-G-G-T-C (which was at a frequency of 0.7% in the EUR group, and is found at 13% frequency in AME) and C-G-G-T-C, which has increased from 36.3% to 42%, while the remaining ε3 haplotype, G-G-A-T-C, has decreased from 41% to 30% in frequency.

The EAS macroarea is also characterized by the same five variants already mentioned (rs440446, rs769449, rs769450, rs429358, and rs7412) with the inclusion of another SNP already linked to neurodegeneration: rs150375400, a polymorphism found, in this dataset, only in the East Asian populations. The minor frequency allele rs150375400-G is found only in association with the risk allele rs440446-G and only in two haplotypes leading to an APOE ε3 isoform (one of them rare). In total, nine combinations emerge: one ε2-carrying haplotype, six sequences with ε3 terminal alleles (two of which rare), and two ε4 haplotypes (one rare). The most frequent combination (56.9%) is the one that does not include any disease-linked allele, C-A-G-G-T-C (ε3 haplotype).

A more complex haplotype emerges for the AFR macroarea (Supplementary Table S6). In this specific case, variant rs769449 is only present in two ACB and seven ASW individuals but not in the other AFR populations. To the remaining four core variants (rs440446, rs769450, rs429358, and rs7412) are added two variants of unknown biological impact, but investigated in association with plasma lipoprotein traits (rs9282609 and rs877973), as well as three variants in complete linkage disequilibrium: rs61357706-A and rs115299243-G have been associated with an increased risk of cardiovascular ailments, while rs769455-T (also known as “ApoE4 Philadelphia” or “ApoE Qatar”) has been studied in AD and is associated with dyslipidemia. The combination of alleles produces nine haplotypes, one of which is rare (present only once). The others are subdivided in one ε2-linked sequence (at a frequency of 10.3%), six ε3 haplotypes (the most frequent being C-G-C-G-A-A-T-C-C at 29.3% and including only risk alleles for Alzheimer’s disease), and one ε4-carrying combination (with a frequency of 26.3%).

## 4. Discussion

The present investigation has been carried out to assess the presence and distribution of APOE haplotypes, or allele sequence combinations, in a large cohort of self-reportedly healthy individual (at time of collection) representatives of worldwide genetic variation, with the idea that a more extensive knowledge of the variability of these sequences across populations and macroareas, especially in terms of haplotypes carrying potentially causative alleles for cardiovascular ailments and neurodegenerative diseases in healthy cohorts, could be supportive for a more adequate study of the affected individuals by including the nuances of population genomics-derived information in a clinical and diagnostic setting.

The distribution of APOE isoforms across macroareas mirrors what is expected according to previous studies with larger cohorts and a more diverse population roster [13]: the African macroarea presents a much higher proportion of the ε2 and ε4 isoforms, as well as a reduction of the ε3 isoform, and the populations that are geographically closer to the Equator have a higher proportion of the ε4 isoform, when compared to the other members of each macroarea. This behavior has been ascribed to a number of possible non-genetic factors and environmental interactions across the lifespan, among which are protective effects against exposure to pathogens (especially in early childhood), preservation of cognitive abilities, and enhanced fertility rates in hostile environments. Similarly, a gradient can be observed in the EUR macroarea, where populations that reside at higher latitudes have a higher proportion of APOE ε4, possibly as a mechanism to improve lipoprotein metabolism in colder environments and counteract the effect of less exposure to UV light and less vitamin D production [13].

**Figure 6:**
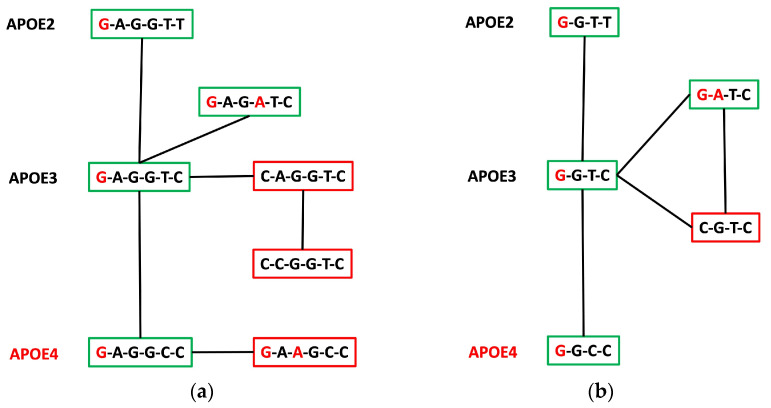
Stepping-stone model for the change from one haplotype to another with a maximum of one mutation. (**a**) Model for the six-variant core haplotype. (**b**) Model for the four-variant super-core haplotype. Green haplotypes are more frequent in the AFR macroarea; red haplotypes are less frequent.

Indeed, our approach has also revealed several levels of haplotype sharing across and inside macroareas, according to which degree of distribution across populations we consider. In general, looking at both core and super-core haplotypes (constituted by variants shared across all macroareas in the first case, and in all populations in the second case), it can be seen that there tends to be a higher proportion of haplotypes bearing non-causative alleles and the ε3 isoform in populations of South Asian and East Asian ancestry, while the African macroarea is enriched in haplotypes carrying the ε2 and ε4 isoforms, as can be expected. Considering the six-variant core haplotype, these populations interestingly tend to have a much higher proportion of haplotypes starting with the AD-risk allele rs440446-G, with an especially high frequency in the ε4-carrying haplotype, which is at 25.5% against a 3% of Europeans with the same sequence (Figure 6). Conversely, Europeans present a higher frequency of the ε4-bearing sequence G-A-A-G-C-C, which carries two AD-risk alleles (rs440446-G in the first position, and rs769449-A in the third position). When looking at the step-by-step frequency graph, it is interesting to notice that it is impossible to find a haplotype that carries all the damaging alleles, while it is much more common overall to come across the one with all neutral ones (C-A-G-G-T-C), which is at high frequencies in all macroareas (56.4% in EAS, 45.7% in SAS, 41.6% in AME, and 36.2% in EUR) except in Africa, where it stands at 11.8%. Indeed, if we consider the possible connecting networks that allow the change from a haplotype to another with one single mutation at a time (Figure 6), we can notice that, given a fixed number of individuals, in order for the G-starting haplotypes to be at higher frequency, the neutral ones have to reduce in frequency as well. This is also maintained in the four-variant super-core haplotype, where the neutral allele-bearing sequence is much less frequent in Africa (11.9%) than the rest of the other haplotypes, especially those carrying ε2 and ε4.

When focusing on haplotypes composed of variants that are polymorphic in all populations for a specific macroarea, but not necessarily in all populations of other macroareas, interesting new aspects emerge. First, one can notice that variant rs769449 appears in all macroarea-delimited haplotypes, apart from the African one, where it shows up as polymorphic only in the admixed populations ACB (Caribbeans of African ancestry) and ASW (Americans of African ancestry in Southwestern USA) and in limited numbers (two and seven individuals, respectively, carry the minor frequency allele). This very interesting observation suggests that the risk allele may have been carried in those populations by contact with European groups or people of local ancestry, rather than the African individuals, who are otherwise monomorphic for the neutral allele (Supplementary Table S1). Similarly, mutation rs150375400-G is uniquely present in individuals of East Asian ancestry, where it was recently reported as a rare variant with possible impact on the risk of mild cognitive impairment (MCI) and AD in a Chinese cohort of 257 individuals (69 diagnosed with AD, 83 with MCI, and 107 healthy controls) [31]. The most interesting observation, however, is that all macroarea-delimited haplotypes include only variants with possible impact on neurodegeneration and cognition, apart from the AFR macroarea, which presents four unique variants (rs9282609, rs877973, rs61357706, and rs115299243) all linked to dyslipidemia and cardiovascular ailments (plus a fifth variant, rs769455, carrying an allele connected to a higher risk of AD [32,33]). Three of these variants, rs61357706, rs115299243, and rs769455, are in high genetic linkage and their risk alleles are always found together in 33 individuals of African ancestry across all seven populations (including the admixed ACB and ASW), as well as three individuals belonging to the AME macroarea. From a population genetics perspective, it seems likely that these mutations have appeared in Africa and have then spread to populations of the American continent, possibly during the historical slave trade in the context of the European colonization of the Americas. More extensive tests with a higher number of subjects may better elucidate how extensive the sharing of mutation-carrying segments is among pairs of individuals and provide an estimate for a date of origin for those mutations. Nonetheless, the data suggest that individuals of African ancestry may be more likely to simultaneously carry multiple variants linked to cardiovascular ailments and lipid metabolism dysfunction in the sequence of APOE, on the background of potentially neurodegenerative alleles, while individuals from other macroareas may predominantly carry mutations related to AD and dementia in different positions along the gene. Indeed, it is very telling that groups of different local ancestry showcase different risk-associated mutations, remarking the necessity to acquire extensive knowledge around putatively causative mutations [34]. Thanks to the relatively recent introduction of paleoanthropology, it has been revealed that several genetic changes associated with adaptive phenotypes and diseases in the current populations have been inherited from ancestral inhabitants of Europe and Asia that were present there before the arrival of our species. Moreover, it is clear that the ancestral history of a population massively impacts its genetic makeup and, consequently, the phenotypes (especially the maladaptive ones) of modern individuals [35,36].

This study, even though based on putatively healthy individuals, suggests that a one-for-all approach to genetic-based diagnostics is inappropriate in the context of complex pathologies and for genes with a pleiotropic function [37,38,39]. Indeed, ancestry-informed screening for specific mutations and haplotypes, which are different for different populations, is required in contemporary medicine, as it may be advantageous especially for individuals from populations of admixed ancestry, as well as for people whose recent ancestors have migrated to a different country.

## Figures and Tables

**Table 1:** List of unique and shared variants across macroareas, with disease involvement.

AFR	AME	EAS	EUR	SAS	Shared
rs72654467	rs528229851	rs373985746 ^2^	rs563571689	rs555840707	rs565782572
rs1187843706	rs538246559	rs192348494 ^2^	rs769447	rs555877419	rs769450 ^2^
rs184686013	rs539807928	rs150375400 ^2^	rs769452 ^3^	rs550501196	rs769449 ^2^
rs375741166	rs535397097	rs373651604	rs186466504	rs555914310	rs440446 ^2^
rs189660912	rs1227709957	rs549553647	rs121918393 ^2^	rs552962455	rs7412 ^4^
rs564144591	rs1313313298	rs533904656 ^3^	rs530010303 ^1^	rs572713679	rs429358 ^4^
rs148558158	rs557715042	rs140808909 ^1^		rs542186645	
rs1368528953		rs190853081 ^3^		rs529662056	
rs577618688		rs553874843 ^1^		rs563103121	
rs1018669382 ^1^				rs555222732	
rs1181840153				rs774452222	
				rs569017773	

^1^ Variant involved in CVD. ^2^ Variant involved in AD. ^3^ Variant involved in CVD and AD. ^4^ Variant defining APOE isoforms.

## Data Availability

Publicly available data from the 1KGP used in the present study can be recovered at http://ftp.1000genomes.ebi.ac.uk/vol1/ftp/data_collections/. All other sources of information have been pointed out in the main text.

## Supplementary Materials

The following supporting information can be downloaded at: https://www.mdpi.com/article/10.3390/cimb45040184/s1, Table S1: Variant identity, numerosity and frequency of the A2 allele across populations; Table S2: Numerosity of the A2 allele and variant uniqueness across macroareas; Table S3: Frequency of core allele combinations across macroareas, and contribution of each macroarea to each haplotype; Table S4: Frequency of super-core haplotypes across populations in each macroarea; Table S5: Frequency of super-core haplotypes across macroareas, and contribution of each macroarea to each haplotype; Table S6: Frequency of macroarea-specific allele combination within each population.
